# A case–control study of infections caused by *Klebsiella pneumoniae* producing New Delhi metallo-beta-lactamase-1: Predictors and outcomes

**DOI:** 10.3389/fcimb.2022.867347

**Published:** 2022-07-28

**Authors:** Eduardo Rodríguez-Noriega, Elvira Garza-González, Paola Bocanegra-Ibarias, Beatriz Alejandra Paz-Velarde, Sergio Esparza-Ahumada, Esteban González-Díaz, Héctor R. Pérez-Gómez, Rodrigo Escobedo-Sánchez, Gerardo León-Garnica, Rayo Morfín-Otero

**Affiliations:** ^1^ Instituto de Patología Infecciosa y Experimental “Dr. Francisco Ruiz Sánchez”, Centro Universitario de Ciencias de la Salud, Guadalajara, Mexico; ^2^ Facultad de Medicina, Universidad Autónoma de Nuevo León, Monterrey, Mexico; ^3^ Hospital Civil de Guadalajara. Epidemiology, ‎Microbiology and Infectious Disease Department, Guadalajara, Mexico

**Keywords:** NDM-1, *Klebsiella pneumoniae*, bacterial resistance, resistance mechanisms, carbapenemases, metallo-beta-lactamases, healthcare-associated infections

## Abstract

**Introduction:**

Infections caused by antimicrobial-resistant bacteria are a significant cause of death worldwide, and carbapenemase-producing bacteria are the principal agents. New Delhi metallo-beta-lactamase-1 producing *Klebsiella pneumoniae* (KP-NDM-1) is an extensively drug-resistant bacterium that has been previously reported in Mexico. Our aim was to conduct a case–control study to describe the risk factors associated with nosocomial infections caused by *K. pneumoniae* producing NDM-1 in a tertiary-care hospital in Mexico.

**Methods:**

A retrospective case–control study with patients hospitalized from January 2012 to February 2018 at the Hospital Civil de Guadalajara “Fray Antonio Alcalde” was designed. During this period, 139 patients with a culture that was positive for *K. pneumoniae* NDM-1 (cases) and 486 patients hospitalized in the same department and on the same date as the cases (controls) were included. Data were analyzed using SPSS v. 24, and logistic regression analysis was conducted to calculate the risk factors for KP-NDM-1 infection.

**Results:**

One hundred and thirty-nine case patients with a KP-NDM-1 isolate and 486 control patients were analyzed. In the case group, acute renal failure was a significant comorbidity, hospitalization days were extended, and significantly more deaths occurred. In a multivariate analysis of risk factors, the independent variables included the previous use of antibiotics (odds ratio, OR = 12.252), the use of a urinary catheter (OR = 5.985), the use of a central venous catheter (OR = 5.518), the use of mechanical ventilation (OR = 3.459), and the length of intensive care unit (ICU) stay (OR = 2.334) as predictors of infection with NDM-1 *K. pneumoniae.*

**Conclusion:**

In this study, the previous use of antibiotics, the use of a urinary catheter, the use of a central venous catheter, the use of mechanical ventilation, and ICU stay were shown to be predictors of infection with NDM-1 *K. pneumoniae* and were independent risk factors for infection with NDM-1 *K. pneumoniae.*

## Introduction

The world population is now under severe threat from bacteria resistant to multiple antimicrobial agents (Antimicrobial Resistance, 2022). Other infectious diseases, such as influenza and coronavirus disease 2019 (COVID-19), have diverted our attention from the ever-growing problem of resistance in the presence of difficult-to-treat infections caused by multidrug-resistant, extensively drug-resistant, and pandrug-resistant bacteria (Magiorakos et al., 2012; Kadri et al., 2018).

The onset of obstacles created by ESKAPE organisms (*Enterococcus faecium*, *Staphylococcus aureus*, *Klebsiella pneumoniae*, *Acinetobacter baumannii*, *Pseudomonas aeruginosa*, and Enterobacter species) and others, and now by bacteria producing New Delhi metallo-beta-lactamase-1 (NDM-1), has increased the urgency (Boucher and Corey, 2008; Rice, 2008; Boucher et al., 2009).

The new NDM-1 was reported as a transmissible genetic element encoding multiple resistance genes isolated from a strain of *Klebsiella* (Yong et al., 2009). This report was followed by a study that detected NDM-1 in *K. pneumoniae* and *Escherichia coli* in India, the United Kingdom, and Pakistan (Kumarasamy et al., 2010). In that study, the isolates were extensively drug-resistant and susceptible only to tigecycline and colistin (Kumarasamy et al., 2010). The rapid, unexpected spread of NDM-1 has forewarned the need for a robust worldwide response to international monitoring, surveillance, and tracking systems (Kumarasamy et al., 2010; Bonomo, 2011).

Presently, NDM-1 has been detected in environmental samples in India, Europe, and Canada, renewing calls for vigilance (Struelens et al., 2010; Walsh et al., 2011; Borgia et al., 2012; Lowe et al., 2013). The expansion of detected NDM-1 continued with *K. pneumoniae* co-producing NDM-1 and KPC-2 (Wu et al., 2019; Gao et al., 2020; Junaid, 2021).

NDM-1 has been discovered in different bacterial species in patients with nosocomial infections related to horizontal transfer and intraspecies spread in Mexico (Duran-Bedolla et al., 2019). The persistent dissemination of diverse NDM-1-producing bacteria in Mexico has caused outbreaks with an endemic pattern punctuated by cyclic outbursts (Bocanegra-Ibarias et al., 2017; Petersen-Morfin et al., 2017; Garza-Gonzalez et al., 2021; Fernandez-Garcia et al., 2022).

Our aim was to conduct a case–control study to describe the risk factors associated with nosocomial infections caused by *K. pneumoniae* producing NDM-1 in a tertiary-care hospital in Mexico.

## Methods

### Study design

A retrospective case–control study with patients hospitalized from January 2012 to February 2018 at the Hospital Civil de Guadalajara “Fray Antonio Alcalde” was designed. This hospital is an 899-bed tertiary-care teaching hospital located in Guadalajara, the second-largest city in Mexico. This hospital provides care to adult and pediatric patients in 31 wards in three connected buildings. During this period, we evaluated 139 patients defined as cases (patients with a culture that was positive for NDM-1) and 486 patients defined as controls (patients hospitalized in the same room and on the same date as the cases). The control patients were selected from the same population as the case patients. This group of patients was admitted during the same period as the case patients and hospitalized in the same hospital service in which the case patients were located; this was done to prevent biased estimates of relative risk that occur when patients with positive cultures for susceptible bacteria are included as a control group (Harris et al., 2001; Harris et al., 2002). We excluded patients who were hospitalized for <48 h.

The CDC/NHSN surveillance definitions of healthcare-associated infections include the following: for skin and soft tissue infections and surgical wound infection, the presence of purulent drainage indicates infection; for blood isolates, bacteria isolated from a blood culture bottle indicates infection; for intra-abdominal infections, a positive culture from purulent material obtained during surgery indicates infection; for urine infections, the presence of fever and positive urine culture indicates infection; and for a positive respiratory specimen, the presence of fever, leukocytosis, increased respiratory secretions, and tachypnea (reference) indicates infection.

Clinical and demographic data were collected from the clinical records of the patients and controls, including data on demographic information, previous hospitalizations, the prior use of antibiotics, and the time of discharge. The Sequential Organ Failure Assessment (SOFA) score, Acute Physiology and Chronic Health Evaluation (APACHE) II score, and Glasgow coma scale were used.

### Microbiological and molecular analysis

Clinical isolates were identified by matrix-assisted laser desorption ionization time-of-flight mass spectrometry using the Bruker Biotyper system (Bruker Daltonics, Germany) as described previously (Levesque et al., 2015).

Antimicrobial susceptibility was determined using the VITEK 2 system (bioMérieux, Marcy l’Etoile, France). Carbapenem-resistant isolates were screened to detect carbapenemase production using the CarbaNP test according to the Clinical and Laboratory Standards Institute (CLSI) guidelines (Hamprecht et al., 2013; CLSI, 2019). For all CarbaNP-positive isolates, DNA was extracted directly from the clinical isolates by thermal lysis, and PCR was conducted to detect the carbapenemase-encoding genes (class A: *bla*_KPC-type_; class B: *bla*_VIM-type_, *bla*_IMP-type_, *bla*_NDM-type_; and class D: *bla*_OXA-48 type_) as previously described (Bocanegra-Ibarias et al., 2019). A selection of positive PCR products was confirmed by sequencing (Bonnin et al., 2012).

Data were analyzed using SPSS (IBM SPSS Statistics, USA) v. 24, and logistic regression analysis was conducted to calculate the odds ratios (ORs). The *t*-test was used for independent variables, and the *χ* ^2^ test was used to evaluate differences between groups. A *p*-value <0.05 was considered significant.

## Results

### Study population

We enrolled 139 patients with KP-NDM-1 infection and 486 control patients (**Supplementary Figure 1**); both groups were similar in terms of sex and age. Furthermore, comorbidities were similar in both groups except for acute renal failure, which was significant in the case group (**Table 1**).

**Table 1:** Characteristics of cases of patients infected with *Klebsiella pneumoniae* NDM-1 and controls.

Variable	Cases *N* = 139 (%)	Controls *N* = 486 (%)	OR	*p*
Gender, males/females	86 (61.9)/53 (38.1)	324 (66.7)/162 (33.3)	1.233	0.171
Age (years)	41.17 ± 22.74	42.04 ± 25.11		0.714
Diabetes mellitus	34 (24.5)	111 (22.8)	1.094	0.384
Hypertension	40 (28.8)	139 (28.6)	1.009	0.523
Neurological disease	59 (42.4)	154 (31.7)	1.590	0.013
Cardiac disease	33 (23.7)	80 (16.5)	1.580	0.035
Lung disease	46 (33.1)	151 (31.1)	1.097	0.361
Liver disease	7 (5)	28 (5.8)	0.867	0.467
Biliary disease	5 (3.6)	25 (5.1)	0.688	0.309
Acute renal failure	54 (38.8)	75 (15.4)	3.481	<0.001*
Chronic renal failure	15 (10.8)	58 (11.9)	0.893	0.421
Transplant	5 (3.6)	8 (1.6)	2.229	0.140
Immunosuppression	21 (15.1)	55 (11.3)	1.395	0.145
Alcoholism	24 (17.3)	125 (25.7)	0.603	0.023
Cancer	6 (4.3)	38 (7.8)	0.532	0.105
Previous hospitalization (6 months)	48 (34.5)	155 (31.9)	1.126	0.313
Previous surgery (1 month)	18 (12.9)	43 (8.8)	1.533	0.103
Glasgow coma scale	11.46 ± 3.9	13.36 ± 3.12		<0.001*
SOFA	4.37 ± 4.08	2.58 ± 3.02		<0.001*
APACHE II score	4.37 ± 4.08	2.58 ± 3.02		<0.001*
Creatinine	1.34 ± 2.02	2.4 ± 9.14		0.201
Alkaline phosphatase	171.8 ± 138.1	123.95 ± 104.4		<0.001*
Procalcitonin	10.31 ± 28.14	5.61 ± 15.08		0.103
C-reactive protein	167.34 ± 94.8	82.83 ± 91.3		<0.001*
Days of hospital stay	43.05 ± 31.3	15.58 ± 14.4		<0.001*
Mortality risk	26.14 ± 19.39	17.97 ± 14.8		<0.001*
Outcome	89 (64)	445 (91.6)	6.098	<0.001*
Improvement Death	50 (36)	41 (8.4)		

Values are expressed as mean ± SD or n (%).

SOFA, Sequential Organ Failure Assessment score; APACHE, Acute Physiology and Chronic Health Evaluation.

*p-value is less than 0.05.

The Glasgow coma scale, SOFA, and APACHE scores were significant in the case group (**Table 1**). Abnormal laboratory parameters in the case group included elevated alkaline phosphatase and C-reactive protein levels (**Table 1**). The number of hospitalization days was prolonged in the case group compared to the control group (43.05 ± 31.3 and 15.58 ± 14.4 days, *p* < 0.001, respectively) (**Table 1**).

There were significantly more deaths in the case group (50, 36%) than in the control group (41, 8.4%) (**Table 1**). Previous antibiotic use, including tigecycline, meropenem, linezolid, and piperacillin/tazobactam, was a significant risk factor (**Table 2**). The sources of *K. pneumoniae* producing NDM-1 were urine, respiratory secretions, blood, and wound secretions (**Table 3**).

**Table 2:** Previous antibiotics used for patients infected with *Klebsiella pneumoniae* NDM-1 and controls.

Variable	Cases *N* = 139 (%)	Controls *N* = 486 (%)	OR	*p*
Previous antibiotics	133 (95.7)	313 (64.4)	12.252	<0.001*
Meropenem	94 (67.6)	53 (10.9)	17.066	<0.001*
Linezolid	72 (51.8)	53 (10.9)	8.779	<0.001*
Piperacillin/tazobactam	45 (32.4)	48 (9.9)	4.368	<0.001*
Tigecycline	61 (43.9)	22 (4.5)	33.770	<0.001*

*p-value is less than 0.05.

**Table 3:** Sources of *Klebsiella pneumoniae* NDM-1 isolates.

Isolation source	No. of isolates (%)
Urine	41 (29.5)
Respiratory sources	28 (20.1)
Blood	28 (20.1)
Wound secretions	28 (20.1)
Intra-abdominal	6 (4.4)
Vascular catheter	4 (2.9)
Cerebrospinal fluid	4 (2.9)

### Multivariate analysis

In a multivariate analysis of risk factors, the independent variables included the use of previous antibiotics (odds ratio, OR = 12.252), the use of a urinary catheter (OR = 5.985), the use of a central venous catheter (OR = 5.518), the use of mechanical ventilation (OR = 3.459), and the length of intensive care unit (ICU) stay (OR = 2.334) as predictors of developing an infection with *K. pneumoniae* NDM-1 (**Table 4**).

**Table 4:** Multivariate analysis of risk factors associated with *Klebsiella pneumoniae* NDM-1 infection.

Variable	OR	95% confidence interval	*p*
Surgery	1.713	1.146–2.560	0.009
Intensive care unit stay	2.334	1.564–3.483	<0.001
Central venous catheter	5.518	3.631–8.385	<0.001
Mechanical ventilation	3.459	2.341–5.109	<0.001
Blood transfusion	2.445	1.664–3.592	<0.001
Urinary catheter	5.985	3.495–10.249	<0.001
Previous antibiotics (any)	12.252	5.296–28.346	<0.001
Previous meropenem	17.066	10.821–26.915	<0.001
Previous linezolid	8.779	5.664–13.608	<0.001
Previous piperacillin/tazobactam	4.368	2.747–6.496	<0.001
Previous tigecycline	33.770	17.019–67.008	<0.001

## Discussion

In our study, the patients had several comorbidities, including diabetes mellitus and other chronic diseases such as hypertension; acute renal failure was associated with increased morbidity, and these patients had longer mean hospital stays.

A recent report from South Africa reported similar findings from a nosocomial outbreak of NDM-1 producers in an adult ICU (de Jager et al., 2015). In this matched case–control study, 38 cases and 68 controls were included; *K. pneumoniae* was the most common NDM-1 producer (28/38, 74%), patients had longer mean hospital stays (44.0 vs. 13.3 days; *p* < 0.001) and ICU stays (32.5 vs. 8.3 days; *p* < 0.001), and comorbidities were significant risk factors in the multivariate analysis (de Jager et al., 2015). In our findings, using a urinary catheter, central venous catheter, and mechanical ventilation and the length of ICU stay in a hospital area were significant risk factors for acquiring KP-NDM-1 infections. The association between the use of medical devices and significant risk factors for obtaining resistant bacteria has been documented.

The relationship among medical devices, antimicrobial resistance, and healthcare-associated infections is regularly analyzed by the Centers for Disease Control and Prevention’s National Healthcare Safety Network. The percentages of pathogens with non-susceptibility were significantly higher among device-associated healthcare-associated infections (Weiner-Lastinger et al., 2020). In a review of previously published studies on risk factors for infection with carbapenem-resistant bacteria with carbapenem resistance, the factors that are more frequently reported included previous antibiotic use (91.1%), previous carbapenem use (82.6%), previous colonization (72.7%), the use of mechanical ventilation (66.7%), previous ICU stay (64.4%), dialysis (61.1%), the use of a catheter (58.0%), hospital length of stay (54.5%), comorbidities (52.7%), APACHE II scores (51.7%), and intubation (51.4%) (Palacios-Baena et al., 2021). Device- and medical procedure-related infections are not exclusive to *K. pneumoniae*; multidrug-resistant *E. coli* can also be involved (Kourtis et al., 2021).

In our case, a significant risk factor for acquiring KP-NDM-1 infection was the use of previous antibiotics, including meropenem, which was analogous to device-related infections; the use of antibiotics has also been reported as a risk factor in the medical literature (Tian et al., 2018; Snyder et al., 2019; Kollef et al., 2021; Seo et al., 2021). Carbapenem-resistant *K. pneumoniae* and KP-NDM-1 can cause outbreaks (Zhu et al., 2020). A report from Singapore analyzing an outbreak of NDM-1-producing *Enterobacter cloacae* among adults admitted to an acute hospital’s general ward showed that comorbidities and recent antibiotic use were significant risk factors (Ho et al., 2016). Three reports from Tuscany, Italy, discussed outbreaks in hospitals in that region. First, a large outbreak of NDM-1-producing *K. pneumoniae* sequence type (ST) 147 occurred, during which time the *K. pneumoniae* isolates changed from NDM-1 to NDM-9 (Falcone et al., 2020).

The Tuscany area has increased the isolation of NDM-producing carbapenem-resistant bacteria, with *K. pneumoniae* ST147/NDM-1 being the dominant clone (Tavoschi et al., 2020). Outbreaks can be prolonged in the region, including those that started in November 2018, continuing in 2020 and throughout 2021 (Martin et al., 2021).

The expeditious dissemination of NDM-producing bacteria is concerning; in 2013, after an outbreak caused by an NDM-producing *E. coli*, researchers developed a simple social network (ego network) to identify patients carrying the bacteria admitted to other hospitals in the region. In the follow-up, 61% of the patients were admitted to different hospitals, and additional NDM cases were reported (Ray et al., 2018). Similarly, a regional outbreak of the *bla*_NDM-1_ ST147 *K. pneumoniae* strain spread across the Chicago area in post-acute care facilities (Lapp et al., 2021). In Singapore, the movement of patients in a healthcare network poses challenges for the control of carbapenemase-producing Enterobacterales; in investigating the risk factors for intra- and interfacility transmission from acute care hospitals and long-term care facilities, investigators found that the odds of carbapenemase-producing Enterobacterales colonization increased significantly with a more extended hospital stay, penicillin use, proton pump inhibitor use, a dementia diagnosis, a connective tissue disease diagnosis, and prior carbapenem-resistant Enterobacterales carriage in acute care hospitals (Aung et al., 2021).

In intermediate- and long-term care facilities, wounds, respiratory procedures, vancomycin-resistant *Enterococcus*, and carbapenem-resistant Enterobacterales were significantly associated with infection (Aung et al., 2021). In Europe, an analysis of sequencing data for 143 *bla*_NDM-1_- and *bla*_OXA-48_-positive *K. pneumoniae* isolates from 13 European national collections and the public domain resulted in the identification of 15 previously undetected multicounty transmission clusters (Ludden et al., 2020). In Spain, five major epidemic clones of NDM-producing *K. pneumoniae* caused five nationwide outbreaks in 8 years (Perez-Vazquez et al., 2019). From 2016 to 2018 in 24 provinces and cities in China, the most prevalent carbapenemase genes were *bla*_KPC-2_ and *bla*_NDM_ among *K. pneumoniae* isolates from adult patients and among *E. coli* isolates from children, respectively (Han et al., 2020).

In a 2015 study in Los Angeles, California, USA, carbapenem-resistant Enterobacteriaceae isolates were found in 10 pediatric patients (Pannaraj et al., 2015). In China, carbapenem-resistant *K. pneumoniae* positive for *bla*NDM-1 was found in 87.2% (41/47) of neonates (Yin et al., 2018). Also, in neonates in China, carbapenem-resistant *K. pneumoniae* carried NDM-5 and NDM-1 (Luo et al., 2021). In Mexico, NDM-1-producing bacteria are not only endemic problems with occasional outbreaks in adult, pediatric, and neonatal ICUs in our hospital but also around the country.

In association with different investigators in Mexico, the rates of antimicrobial resistance were first studied in samples from 47 centers in 20 states, and carbapenem resistance was detected in 3% of *E. coli*, in 12.5% of *Klebsiella* spp. and *Enterobacter* spp., and in up to 40% of *P. aeruginosa* (Garza-Gonzalez et al., 2019). A second study provided further evidence that resistance to antimicrobial agents is increasing around the country, especially in the *A. baumannii* complex, where high drug resistance has been detected for almost all antibiotics, including carbapenems (Garza-Gonzalez et al., 2020). In the third investigation, phenotypic and genetic data were analyzed. Among Enterobacterales, the most frequently detected carbapenemase-encoding gene was *bla*_NDM-1_ (81.5%), followed by *bla*_OXA-232_ (14.8%) and *blaOXA-181* (7.4%); that in *A. baumannii* was *bla*_OXA-24_ (76%) and that in *P. aeruginosa* was *bla*_IMP_ (25.3%), followed by *bla*_GES_ and *bla*_VIM_ (13.1% each) (Garza-Gonzalez et al., 2021).

Among *K. pneumoniae* isolates, *bla*_TEM_, *bla*_SHV_, and *bla*_CTX_ were detected in 68.79%, 72.3%, and 91.9% of isolates, respectively, and among *E. coli* isolates, *bla*_TEM_, *bla*_SHV_, and *bla*_CTX_ were detected in 20.8%, 4.53%, and 85.7% of isolates, respectively (Garza-Gonzalez et al., 2021). Other reports suggest that NDM-1 is the most frequent carbapenemase-encoding gene in Mexico (Alcantar-Curiel et al., 2019; Toledano-Tableros et al., 2021).

Carbapenemases have spread in Latin America and the Caribbean, with endemic patterns in Brazil, Colombia, Argentina, and Mexico (Escandon-Vargas et al., 2017). In Peru, carbapenem-resistant *K. pneumoniae* isolates were recovered from adults and children with severe bacteremia in a Peruvian hospital in June 2018. All isolates carried *bla*_NDM-1_; most isolates belonged to the ST348 sequence type (Pons et al., 2020). In Argentina, in a 4-year surveillance study, 40 *Acinetobacter* strains carried NDM-1, most of which were *A. baumannii* strains (Adams et al., 2020).

In northern (Amazon region) Brazil, in different healthcare institutions, multidrug-resistant *K. pneumoniae* isolates were found to have *bla*_NDM-7_ (60.9%—14/23) and *bla*_NDM-1_ (34.8%—8/23) variants (Rodrigues et al., 2021).

While the prevalence of carbapenemase-producing bacteria has declined substantially in New York City in recent years, increased detection in patients with COVID-19 may signal the re-emergence of these highly resistant pathogens in the wake of the global pandemic (Gomez-Simmonds et al., 2021).

In France, an outbreak of *K. pneumoniae* producing the carbapenemase NDM-1 occurred in our ICU during the last COVID-19 wave; 12 patients tested positive, seven remained asymptomatic, and five developed an infection (Amarsy et al., 2021). Appropriate surveillance is crucial to control the dissemination of NDM-producing bacteria.

National surveillance programs are crucial; in the Netherlands, Dutch medical microbiology laboratories are asked to submit suspected carbapenemase-producing bacteria to the National Institute for Public Health and the Environment as part of a national surveillance system; the predominant carbapenemase alleles are *bla*_OXA-48_ and *bla*_NDM-1_ (van der Zwaluw et al., 2020).

In Switzerland, from the isolates submitted to the Swiss National Reference Center for Emerging Antibiotic Resistance for 2 years (January 2019–December 2020), among the 108 sequenced isolates, NDM-1 was the most prevalent variant, occurring in 56 isolates, mostly *K. pneumoniae* isolates (34/56); the following most prevalent variant was NDM-5, which occurred in 49 isolates, mainly *E. coli* isolates (40/49) (Findlay et al., 2021).

We are now in a difficult period in world history: the COVID-19 pandemic is occurring simultaneously with the antibiotic resistance crisis. Despite the burden on the medical profession caused by the pandemic, efforts to better treat antibiotic-resistant infections must continue (Chambers et al., 2021; Nieuwlaat et al., 2021). We will need more robust and efficient permanent antimicrobial stewardship at all care levels and better strategies for selecting appropriate antibiotic therapies for difficult-to-treat infections, ranking methodology approaches and timing, the duration of antibiotic infusion, and de-escalation for antibiotic optimization in ICUs (Kadri et al., 2018; Kollef et al., 2021; Okeah et al., 2021; Perez et al., 2021; Tartof et al., 2021; Wilson et al., 2021).

Practical, non-pharmaceutical interventions (handwashing, wearing masks) superbly controlled the 1918 influenza pandemic and the COVID-19 pandemic, and these can now be of value in containing the spread of NDM-1-producing bacteria.

Our study has several limitations, including the lack of information regarding a) the evolution of infection after starting a new antibiotic therapy, b) the treatment for the condition, c) the travel history of the cases, and d) the colonization status.

In conclusion, our study showed that *K. pneumoniae* NDM-1 is more frequent in the study population than other carbapenemase-encoding genes that are more frequent in other populations, such as KPC. There are few therapeutic alternatives for the treatment of bacterial species positive for NDM, rendering them serious threats. In our study, the previous use of antibiotics, the use of a urinary catheter, the use of a central venous catheter, the use of mechanical ventilation, and the length of ICU stay were detected as predictors of developing an infection with *K. pneumoniae* NDM-1.

## Data availability statement

The original contributions presented in the study are included in the article/**supplementary material**. Further inquiries can be directed to the corresponding author.

## Ethics statement

The local ethics committee (Comité de Ética en Investigación del Antiguo Hospital Civil de Guadalajara “Fray Antonio Alcalde,” Jalisco, Mexico) approved this study (reference 003/16). Informed consent was waived by the Ethics Committee because no intervention was involved and no patient-identifying information was included.

## Author contributions

ERN, EGG, and RMO conceived the study design. EGG, PBI, BPV, RMO, ERN, SEA, EGD, HPG, RES, GLG, and EGG contributed the tools and performed the data analysis. ERN, EGG, and RMO drafted the manuscript. All authors contributed to the article and approved the submitted version.

## Conflict of interest

The authors declare that the research was conducted in the absence of any commercial or financial relationships that could be construed as a potential conflict of interest.

## Publisher’s note

All claims expressed in this article are solely those of the authors and do not necessarily represent those of their affiliated organizations, or those of the publisher, the editors and the reviewers. Any product that may be evaluated in this article, or claim that may be made by its manufacturer, is not guaranteed or endorsed by the publisher.
