# Supplementary material for: A case–control study of infections caused by Klebsiella pneumoniae producing New Delhi metallo-beta-lactamase-1: Predictors and outcomes
Source: Front Cell Infect Microbiol. 2022 Jul 28;12:867347. doi: 10.3389/fcimb.2022.867347 (PMC9366880; doi:10.3389/fcimb.2022.867347)
Supplement: Supplementary file 1 [file Presentation_1.pptx]

## Slide 1
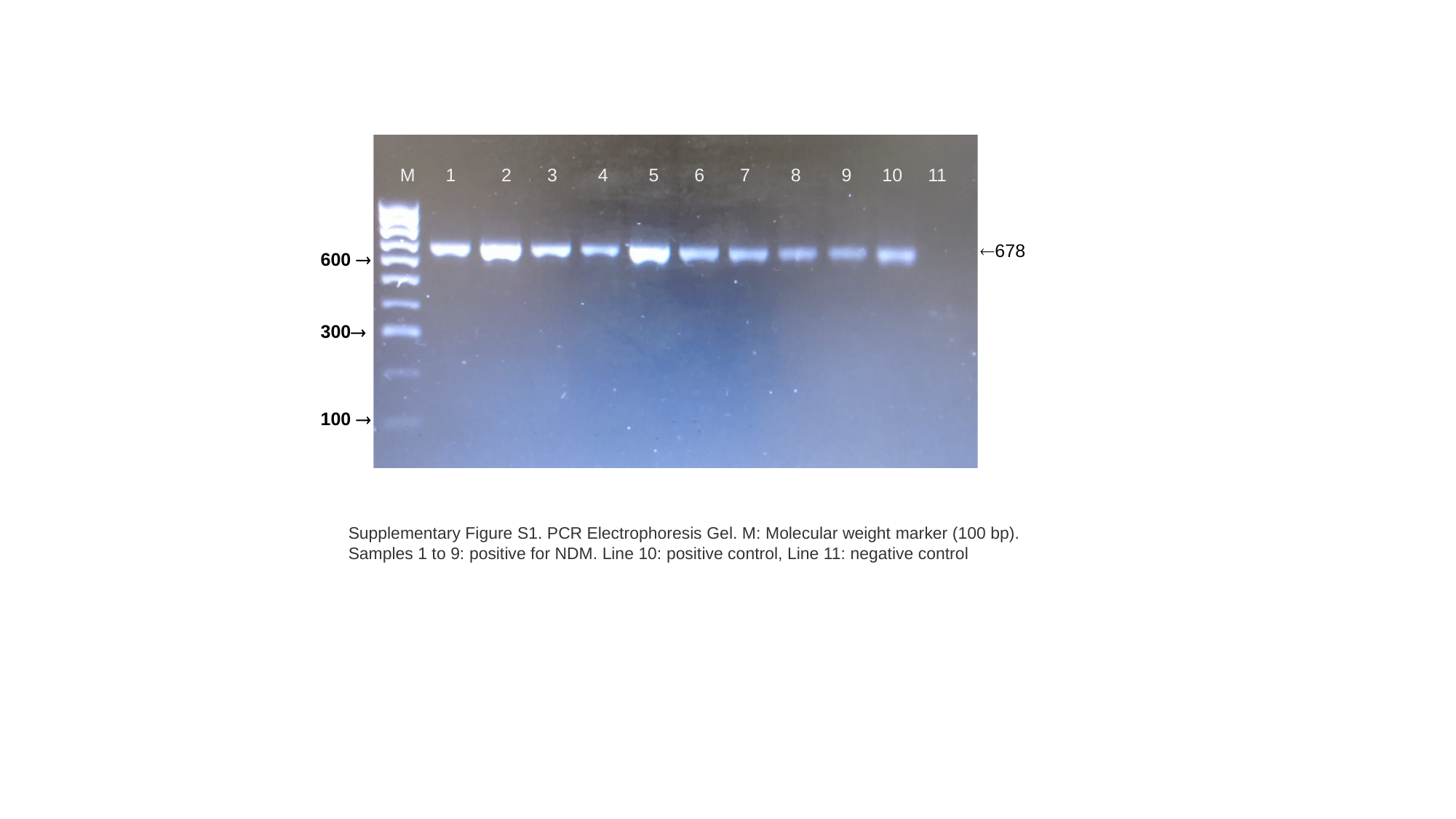

M 1 2 3 4 5 6 7 8 9 10 11
678
600 
300
100 
Supplementary Figure S1. PCR Electrophoresis Gel. M: Molecular weight marker (100 bp). Samples 1 to 9: positive for NDM. Line 10: positive control, Line 11: negative control
