# Supplementary material for: A case–control study of infections caused by Klebsiella pneumoniae producing New Delhi metallo-beta-lactamase-1: Predictors and outcomes
Source: Front Cell Infect Microbiol. 2022 Jul 28;12:867347. doi: 10.3389/fcimb.2022.867347 (PMC9366880; doi:10.3389/fcimb.2022.867347)
Supplement: Supplementary file 2 [file Table_1.docx]

Supplementary table S1, MIC values for antimicrobials tested against KP-NDM-1 isolates.

| **N° Isolate NDM positive** | **Ceftriaxone** | **Ciprofloxacin** | **Levofloxacin** | **Amikacin** | **Meropenem** | **Tigecycline** |
| --- | --- | --- | --- | --- | --- | --- |
| 1 | ≥32 | 1 | NA | ≤2 | ≥8 | 1 |
| 2 | ≥32 | ≥2 | ≥4 | >32 | ≥8 | NA |
| 3 | ≥32 | ≥2 | NA | ≥32 | ≥8 | ≥4 |
| 4 | ≥32 | ≥2 | NA | >32 | ≥8 | ≥4 |
| 5 | ≥32 | ≥2 | NA | >32 | ≥8 | NA |
| 6 | ≥32 | ≥2 | NA | >32 | ≥8 | 2 |
| 7 | NA | 1 | NA | 32 | ≥8 | 1 |
| 8 | ≥32 | 1 | NA | 16 | ≥8 | 1 |
| 9 | ≥32 | ≥2 | NA | >32 | ≥8 | NA |
| 10 | NA | ≥2 | NA | ≥32 | ≥8 | 2 |
| 11 | NA | ≥2 | NA | ≥32 | ≥8 | ≥4 |
| 12 | ≥32 | ≥2 | NA | ≥32 | ≥8 | ≥4 |
| 13 | NA | ≥2 | NA | ≥32 | ≥8 | ≥4 |
| 14 | NA | ≥2 | NA | ≥32 | ≥8 | ≥4 |
| 15 | ≥32 | ≥2 | ≥4 | >32 | ≥8 | NA |
| 16 | ≥32 | NA | ≥4 | NA | ≥8 | 2 |
| 17 | NA | NA | ≥4 | NA | ≥8 | ≥4 |
| 18 | ≥32 | NA | ≥4 | NA | ≥8 | NA |
| 19 | NA | ≥2 | NA | ≥32 | ≥8 | ≥4 |
| 20 | ≥32 | NA | ≥4 | NA | ≥8 | NA |
| 21 | ≥32 | NA | ≥4 | NA | ≥8 | NA |
| 22 | ≥32 | ≥2 | NA | ≥32 | ≥8 | ≥4 |
| 23 | NA | NA | ≥4 | NA | ≥8 | ≥4 |
| 24 | ≥32 | NA | ≥4 | NA | ≥8 | NA |
| 25 | NA | NA | ≥4 | NA | ≥8 | ≥4 |
| 26 | ≥32 | NA | ≥4 | NA | ≥8 | NA |
| 27 | ≥32 | NA | ≥4 | NA | ≥8 | ≥4 |
| 28 | ≥32 | NA | ≥4 | NA | ≥8 | 2 |
| 29 | ≥32 | ≥2 | NA | >32 | ≥8 | NA |
| 30 | ≥32 | ≥2 | NA | >32 | ≥8 | NA |
| 31 | ≥32 | ≤0.25 | ≤0.12 | >32 | ≥8 | 1 |
| 32 | ≥32 | NA | ≥4 | NA | ≥8 | 1 |
| 33 | ≥32 | ≥2 | ≥4 | ≤2 | ≥8 | 2 |
| 34 | ≥32 | NA | ≥4 | NA | ≥8 | 2 |
| 35 | ≥32 | ≥2 | ≥4 | >32 | ≥8 | NA |
| 36 | ≥32 | NA | ≥4 | NA | ≥8 | 2 |
| 37 | ≥32 | ≥2 | ≥4 | ≤2 | ≥8 | 2 |
| 38 | ≥32 | ≥2 | ≥4 | ≤2 | ≥8 | 2 |
| 39 | ≥32 | ≥2 | ≥4 | ≤2 | ≥8 | 2 |
| 40 | ≥32 | ≥2 | ≥4 | 4 | ≥8 | 2 |
| 41 | NA | ≥2 | NA | ≤2 | ≥8 | 2 |
| 42 | NA | NA | ≥4 | NA | ≥8 | 1 |
| 43 | ≥32 | NA | NA | NA | ≥8 | 2 |
| 44 | ≥32 | ≥2 | ≥4 | ≤2 | ≥8 | NA |
| 45 | NA | NA | ≥4 | NA | ≥8 | 2 |
| 46 | NA | NA | ≥4 | NA | ≥8 | 2 |
| 47 | ≥32 | ≥2 | ≥4 | ≤2 | ≥8 | NA |
| 48 | NA | NA | ≥4 | NA | ≥8 | 2 |
| 49 | ≥32 | ≥2 | ≥4 | ≤2 | ≥8 | 2 |
| 50 | ≥32 | ≥2 | 1 | >32 | ≥8 | 1 |
| 51 | ≥32 | ≥2 | ≥4 | ≤2 | ≥8 | ≥4 |
| 52 | ≥32 | 1 | 1 | ≤2 | ≥8 | 1 |
| 53 | ≥32 | 1 | 1 | >32 | ≥8 | ≤0.05 |
| 54 | ≥32 | ≥2 | ≥4 | ≤2 | ≥8 | ≥4 |
| 55 | NA | NA | ≥4 | NA | ≥8 | ≥4 |
| 56 | ≥32 | ≥2 | ≥4 | ≤2 | ≥8 | ≥4 |
| 57 | ≥32 | ≥2 | NA | >32 | ≥8 | NA |
| 58 | ≥32 | ≥2 | NA | >32 | ≥8 | NA |
| 59 | ≥32 | ≤0.25 | ≤0.12 | >32 | ≥8 | 1 |
| 60 | NA | NA | ≥4 | NA | ≥8 | ≥4 |
| 61 | ≥32 | NA | ≥4 | NA | ≥8 | NA |
| 62 | ≥32 | ≤0.25 | ≤0.12 | ≤2 | ≥8 | ≤0.05 |
| 63 | ≥32 | NA | ≥4 | NA | ≥8 | NA |
| 64 | ≥32 | ≥2 | ≥4 | >32 | ≥8 | NA |
| 65 | NA | ≥2 | ≥4 | 8 | ≥8 | 2 |
| 66 | ≥32 | NA | ≥4 | NA | ≥8 | NA |
| 67 | ≥32 | NA | ≥4 | NA | ≥8 | NA |
| 68 | ≥32 | NA | ≥4 | NA | ≥8 | NA |
| 69 | ≥32 | ≤0.25 | ≤0.12 | >32 | ≥8 | 1 |
| 70 | ≥32 | NA | 1 | NA | ≥8 | ≤0.05 |
| 71 | ≥32 | NA | ≥4 | NA | ≥8 | 2 |
| 72 | ≥32 | NA | ≥4 | NA | ≥8 | 2 |
| 73 | ≥32 | ≥2 | NA | >32 | ≥8 | ≥4 |
| 74 | ≥32 | ≥2 | NA | ≤2 | ≥8 | NA |
| 75 | ≥32 | ≤0.25 | NA | >32 | ≥8 | ≤0.05 |
| 76 | ≥32 | ≥2 | NA | ≤2 | ≥8 | 2 |
| 77 | ≥32 | 0.5 | 1 | 32 | ≥8 | ≤0.05 |
| 78 | ≥32 | 1 | NA | >32 | ≥8 | 1 |
| 79 | ≥32 | ≥2 | NA | >32 | ≥8 | NA |
| 80 | ≥32 | ≤0.25 | NA | >32 | ≥8 | 1 |
| 81 | ≥32 | ≤0.25 | NA | >32 | ≥8 | 2 |
| 82 | ≥32 | ≥2 | NA | >32 | ≥8 | 2 |
| 83 | ≥32 | 1 | 1 | >32 | ≥8 | 1 |
| 84 | ≥32 | NA | ≥4 | NA | ≥8 | ≥4 |
| 85 | 8 | ≤0.25 | NA | >32 | ≥8 | ≥4 |
| 86 | ≥32 | ≥2 | ≥4 | 16 | ≥8 | NA |
| 87 | ≥32 | NA | ≥4 | NA | ≥8 | ≤0.05 |
| 88 | ≥32 | ≥2 | NA | 4 | ≥8 | NA |
| 89 | ≥32 | ≥2 | ≥4 | 4 | ≥8 | ≥4 |
| 90 | ≥32 | ≥2 | NA | 4 | ≥8 | ≥4 |
| 91 | ≥32 | ≤0.25 | NA | >32 | ≥8 | ≤0.05 |
| 92 | ≥32 | ≤0.25 | NA | >32 | ≥8 | 1 |
| 93 | 8 | ≤0.25 | NA | >32 | ≥8 | ≥4 |
| 94 | ≥32 | ≤0.25 | NA | >32 | ≥8 | ≤0.05 |
| 95 | ≥32 | NA | 1 | NA | ≥8 | 1 |
| 96 | ≥32 | ≤0.25 | NA | >32 | ≥8 | ≤0.05 |
| 97 | ≥32 | ≥2 | NA | ≤2 | ≥8 | ≥4 |
| 98 | ≥32 | ≥2 | NA | 16 | ≥8 | ≥4 |
| 99 | NA | NA | ≥4 | NA | ≥8 | ≥4 |
| 100 | ≥32 | ≥2 | NA | 4 | ≥8 | NA |
| 101 | NA | NA | ≥4 | NA | ≥8 | ≥4 |
| 102 | ≥32 | ≥2 | NA | ≤2 | ≥8 | ≥4 |
| 103 | ≥32 | NA | ≥4 | NA | ≥8 | NA |
| 104 | ≥32 | ≥2 | ≥4 | >32 | ≥8 | NA |
| 105 | ≥32 | ≤0.25 | NA | >32 | ≥8 | 2 |
| 106 | ≥32 | ≥2 | NA | 8 | ≥8 | NA |
| 107 | ≥32 | ≥2 | ≥4 | >32 | ≥8 | ≥4 |
| 108 | ≥32 | ≤0.25 | NA | >32 | ≥8 | ≤0.05 |
| 109 | 16 | ≤0.25 | NA | >32 | ≥8 | ≥4 |
| 110 | ≥32 | ≥2 | 1 | >32 | ≥8 | NA |
| 111 | ≥32 | ≤0.25 | 1 | >32 | ≥8 | NA |
| 112 | ≥32 | ≥2 | NA | 16 | ≥8 | NA |
| 113 | ≥32 | ≥2 | NA | >32 | ≥8 | 1 |
| 114 | ≥32 | ≤0.25 | NA | >32 | ≥8 | ≤0.05 |
| 115 | ≥32 | ≥2 | ≥4 | >32 | ≥8 | NA |
| 116 | ≥32 | ≥2 | NA | ≤2 | ≥8 | NA |
| 117 | ≥32 | ≥2 | ≥4 | >32 | ≥8 | NA |
| 118 | ≥32 | NA | ≥4 | NA | ≥8 | ≥4 |
| 119 | ≥32 | ≥2 | NA | 4 | ≥8 | ≥4 |
| 120 | ≥32 | ≥2 | NA | >32 | ≥8 | NA |
| 121 | ≥32 | ≥2 | ≥4 | >32 | ≥8 | ≥4 |
| 122 | ≥32 | ≤0.25 | NA | >32 | ≥8 | ≤0.05 |
| 123 | ≥32 | ≥2 | NA | 4 | ≥8 | NA |
| 124 | ≥32 | ≥2 | NA | ≤2 | ≥8 | NA |
| 125 | 16 | NA | ≥4 | NA | ≥8 | ≥4 |
| 126 | ≥32 | ≥2 | NA | >32 | ≥8 | 1 |
| 127 | ≥32 | ≥2 | NA | ≤2 | ≥8 | ≥4 |
| 128 | ≥32 | 1 | NA | >32 | ≥8 | ≥4 |
| 129 | ≥32 | ≥2 | ≥4 | ≤2 | ≥8 | NA |
| 130 | ≥32 | ≥2 | NA | ≤2 | ≥8 | ≥4 |
| 131 | ≥32 | ≥2 | NA | ≤2 | ≥8 | ≤0.05 |
| 132 | ≥32 | ≥2 | ≥4 | >32 | ≥8 | ≥4 |
| 133 | 8 | ≥2 | NA | >32 | ≥8 | ≥4 |
| 134 | ≥32 | 1 | NA | >32 | ≥8 | ≥4 |
| 135 | ≥32 | 1 | NA | >32 | ≥8 | ≥4 |
| 136 | ≥32 | ≥2 | NA | >32 | ≥8 | 1 |
| 137 | ≥32 | NA | ≥4 | NA | ≥8 | 2 |
| 138 | ≥32 | ≤0.25 | NA | >32 | ≥8 | ≤0.05 |
| 139 | ≥32 | ≤0.25 | NA | >32 | ≥8 | ≤0.05 |

NA: Not available
